# Performance Improvement of the Acrylic Acid–Polyvinyl Alcohol Gel Dosimeter with an Organic Additive for Radiation Oncology Applications

**DOI:** 10.3390/gels12020176

**Published:** 2026-02-17

**Authors:** Belal Moftah, Khalid A. Rabaeh, Akram A. Moussa, Abdullah S. Bani Issa, Md A. Al Kafi

**Affiliations:** 1 Medical and Clinical Affairs, King Faisal Specialist Hospital and Research Centre, Medinah 42523, Saudi Arabia; 2 Medical Physics Unit, McGill University, Montreal, QC H3A 0G4, Canada; 3 Medical Imaging Department, Faculty of Applied Medical Sciences, The Hashemite University, Zarqa 13133, Jordan; khalidr@hu.edu.jo; 4 Biomedical Physics Department, King Faisal Specialist Hospital and Research Centre, Riyadh 11211, Saudi Arabia; akramalmoussa@gmail.com (A.A.M.); mal-kafi@kfshrc.edu.sa (M.A.A.K.); 5 Physics Department, Faculty of Science, Yarmouk University, Irbid 21163, Jordan; ashbaniissa@gmail.com

**Keywords:** polyvinyl alcohol (PVA), glucose, dosimetry, NMR, radiation, radiotherapy

## Abstract

This study reports the first preparation and characterization of an acrylic acid–glucose–polyvinyl alcohol (ACAGLPVA) polymer gel dosimeter incorporating glucose as an organic additive for radiation oncology applications. Five formulations with glucose concentrations of 0, 10, 20, 25, and 30 wt% were irradiated using a 6-MV photon beam at doses of 0–60 Gy, and the transverse relaxation rate (R_2_) was measured by nuclear magnetic resonance (NMR) relaxometry. The optimal formulation (25 wt% glucose) demonstrated an excellent linear dose response between 0 and 30 Gy (R^2^ = 0.9979) with a sensitivity of 0.177 s^−1^ Gy^−1^, followed by a non-linear response at 30–60 Gy. The dosimeter exhibited dose rate independence (200–600 cGy/min), energy independence (6–15 MV), temperature independence (5–35 °C), and post-irradiation stability for at least 7 days. These characteristics demonstrate the potential of ACAGLPVA gel dosimeters for accurate three-dimensional dose verification in modern radiotherapy applications.

## 1. Introduction

In the field of radiotherapy, achieving precise and accurate radiation dose delivery to tumors is of paramount importance. It is essential not only to effectively target cancerous tissues but also to minimize exposure to surrounding healthy tissues to reduce the risk of side effects and improve patient outcomes. Advances in radiotherapy techniques, such as intensity-modulated radiation therapy (IMRT) and image-guided radiation therapy (IGRT), have enhanced our ability to focus radiation more accurately. However, these advancements necessitate equally sophisticated dosimetry methods to verify and ensure the accuracy of delivered doses. As treatment protocols become more sophisticated, the role of dosimetry in quality assurance becomes increasingly critical. To support this, two-dimensional (2D) film dosimeters [1] and three-dimensional (3D) gel dosimeters have been developed for dose distribution verification and for improving treatment planning systems [2,3,4]. Three-dimensional dosimeters are particularly valuable in advanced techniques requiring steep dose gradients, such as intensity-modulated radiation therapy, volumetric modulated arc therapy, and stereotactic radiosurgery [5,6,7]. Compared with traditional tools like ionization chambers, thermoluminescent dosimeters, diode arrays, and films, 3D gel dosimeters provide superior spatial accuracy in mapping dose distributions [8,9,10,11]. Polymer gel dosimeters, including BANANA (acrylamide, N,N-methylene-bisacrylamide monomers, nitrous oxide, and agarose) and BANG (acrylamide, N,N-methylene-bisacrylamide monomers, nitrous oxide, and gelatin) formulations, coupled with MRI, have become effective methods for verifying 3D dose distributions [12,13]. These overcome the main drawback of Fricke gels, where ferric ion diffusion after irradiation degrades spatial resolution [14,15,16,17,18]. Polymer gel dosimeters operate on radiation-induced structural changes within the gel matrix, including disintegration, re-entanglement, and cross-linking of molecules, which depend on the absorbed dose [19]. These physical transformations can be quantified using one-dimensional scanning techniques such as nuclear magnetic resonance (NMR) [20] and UV-Vis spectrophotometry [21], or three-dimensional imaging modalities including MRI [22,23,24,25], X-ray computed tomography (CT) [26,27,28,29], optical computed tomography (OCT) [30,31,32], Raman spectroscopy, and ultrasound spectroscopy [33,34]. In addition to their high spatial accuracy in dose distribution recording, polymer gel dosimeters are tissue-equivalent and insensitive to radiation direction, making them highly suitable for radiotherapy applications [35,36]. Fabrication involves dissolving a monomer in a gel matrix with a cross-linking agent [37,38,39]. When irradiated in an oxygen-free environment, the monomer undergoes polymerization, and the gel matrix retains the spatial dose distribution without diffusion blurring [40,41]. Irradiated samples become increasingly opaque with dose, and absorbance—arising from polymer microparticles—can be measured using optical methods such as UV–Vis spectrophotometry to correlate absorbance with polymer density and, ultimately, absorbed dose. For example, Rabaeh and Eyadeh (2023) [21] applied UV–Vis spectrophotometry at 500 nm to analyze N-(3-methoxypropyl) acrylamide (NMPA) polymer gels. Absorbance increased linearly with dose between 2 and 20 Gy, and higher co-monomer concentrations (6–8 wt%) improved sensitivity, resulting in greater polymerization. Furthermore, polymerization alters the magnetic relaxation behavior of surrounding water molecules (R_2_ = 1/T_2_), enabling NMR and MRI to provide an additional means of assessing dose response in polymer gel dosimeters.

The performance of polymer gel dosimeters depends strongly on their composition; hence, numerous formulations have been modified to improve accuracy in 3D dose distribution measurements [42]. Several studies have demonstrated that incorporating additional materials into the base gel matrix can enhance sensitivity and stability while preserving independence from energy and dose rate. A major advancement in this regard has been the inclusion of inorganic salts [3,43,44,45,46,47,48,49] and organic additives [7], which promote free radical generation during irradiation and thus yield a stronger and more reliable dose response compared with traditional formulations. For instance, Abtahi et al. (2014) [37] examined the impact of adding glucose and urea to an acrylamide-based polymer gel (PAGAT). Dose sensitivity was assessed using a MAGNETOM Avanto, 1.5 T MRI scanner (Siemens Healthineers, Erlangen, Germany), with additional optical measurements obtained via a double-beam spectrophotometer. The results showed that incorporating 8.5% glucose and 3% urea significantly enhanced dose sensitivity, with the R_2_-dose response reaching up to 2.6 times that of conventional PAGAT gels. It was also observed that dose–absorbance measurements provided a more sensitive approach, with sensitivity influenced by the selected wavelength. Thus, choosing the appropriate wavelength based on the readout system’s capacity is a key parameter in 3D dose measurement. In addition, the incorporation of glucose and urea was found to extend the stability of both irradiated and non-irradiated samples. Eyadeh et al. (2023) [50] reported enhanced dose sensitivity of N-(Hydroxymethyl) acrylamide polymer gel dosimeters through the addition of glucose. Using a 0.5 T NMR system to evaluate the R_2_-dose response, they demonstrated sensitivity improvements of 53%, 68%, 89%, and 115% with 10, 15, 20, and 25 wt% glucose, respectively. The gels exhibited excellent linearity up to 8 Gy and stability for up to 10 days post-irradiation. The study concluded that glucose acts as a sensitizer by promoting free radical generation after irradiation, thereby accelerating monomer polymerization, enhancing dose sensitivity, and extending the linear response range.

Recently, our group developed a novel polymer PVA gel dosimeter (ACAPHG), formulated with PVA as the gel matrix, glutaraldehyde (GTA) as a crosslinker, acrylic acid (ACA) as the monomer, N,N′-methylene-bis-acrylamide (BIS) as the co-monomer, tetrakis(hydroxymethyl)phosphonium chloride (THPC) as an antioxidant, and magnesium chloride (MgCl_2_) as an inorganic sensitizer [51]. The gels were irradiated with doses up to 50 Gy using a 6 MV photon beam from a medical linear accelerator at a dose rate of 600 cGy/min. Dose response was evaluated by UV–VIS spectrophotometry at 630 nm for both irradiated and non-irradiated samples. Results showed a linear increase in optical absorbance with dose up to 30 Gy, with a sensitivity of 0.013 Gy^−1^ s^−1^ for the ACAPHG dosimeter. Furthermore, the performance of the PVA gel dosimeter (ACAPHG) in three dimensions was evaluated using the CyberKnife robotic radiotherapy system and OCT [52]. The results showed a 94.1% gamma pass rate in 2D analysis and a 99% pass rate in 3D analysis.

While glucose has demonstrated effectiveness as a sensitizer in acrylamide-based polymer gel dosimeters [37,50], its potential in PVA-based gel systems remains unexplored. Existing PVA-based gel dosimeters have primarily utilized inorganic sensitizers such as magnesium chloride or calcium chloride [51,52]. This study addresses this gap by presenting the first acrylic acid–PVA dosimeter incorporating glucose as an organic sensitizer. In contrast to traditional polymer gels that cannot set at room temperature and must be refrigerated, the ACAGLPVA formulation sets at room temperature after approximately 10 h of preparation, eliminating the need for refrigeration and simplifying clinical handling. We hypothesize that this combination will yield high dose sensitivity, extended linear response range, and independence from dose rate, beam energy, and irradiation temperature. This work evaluates the dose response, sensitivity, dose rate independence, beam energy independence, temporal stability, and temperature dependence of the ACAGLPVA gel dosimeter for radiation oncology applications.

## 2. Results and Discussion

**Figure 1:**
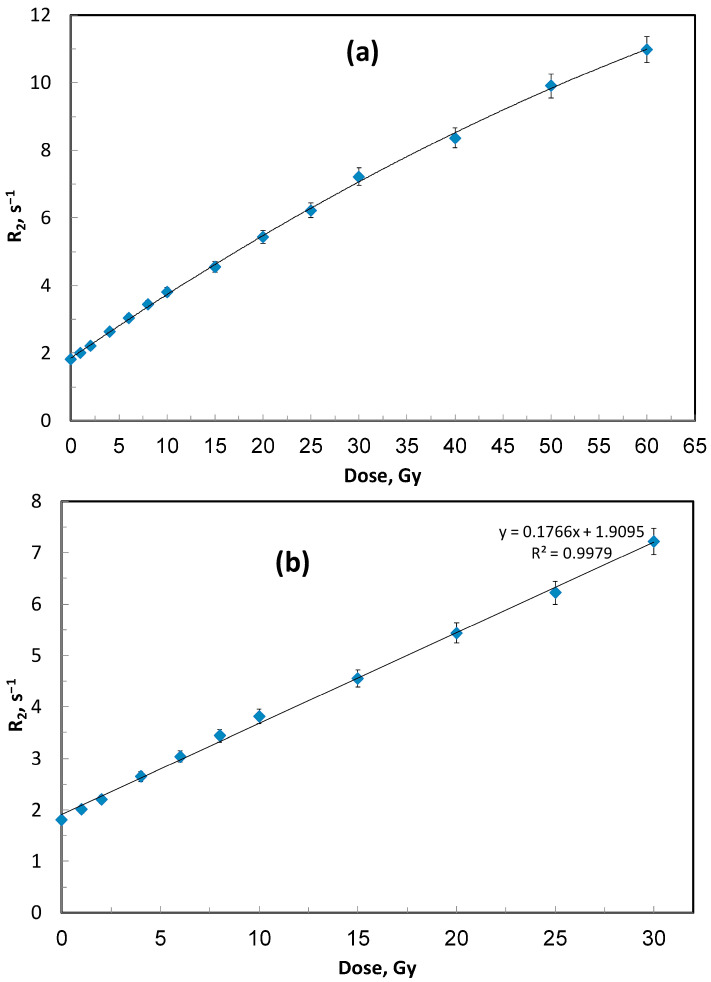
Dose–response relationship of the ACAGLPVA gel dosimeter measured by NMR relaxometry: (**a**) transverse relaxation rate (R_2_) as a function of absorbed dose up to 60 Gy, showing linear response from 0 to 30 Gy followed by non-linear behavior at higher doses; (**b**) linear region (0–30 Gy) with linear fit equation demonstrating excellent linearity and a sensitivity of 0.177 s^−1^ Gy^−1^. Error bars represent 2σ of R_2_ values from three independent samples.

**Figure 2:**
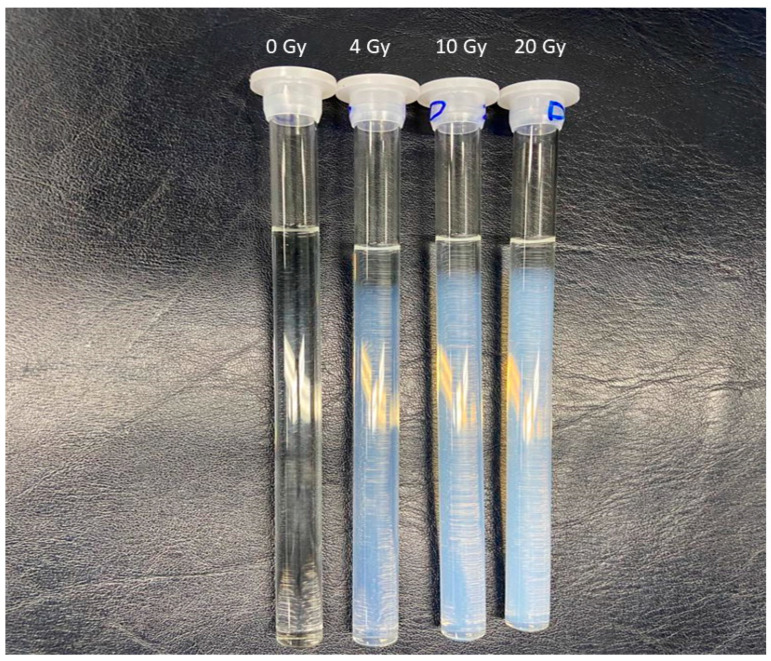
Visual demonstration of radiation-induced polymerization in ACAGLPVA gel dosimeter. Photograph shows gel samples in NMR tubes after irradiation at doses of 4, 10, and 20 Gy (**left** to **right**), alongside a non-irradiated control sample (far **left**). The progressive increase in opacity and color intensity with absorbed dose reflects the formation of polymer microparticles within the PVA gel matrix. Note that the top surface layer (~1–2 mm) shows minimal color change due to oxygen inhibition from the air headspace (see text for details).

The dose–response behavior of the ACAGLPVA polymer gel dosimeter up to 60 Gy is shown in Figure 1 and Figure 2, with sensitivity determined from the linear region (0–30 Gy). The R_2_ values increased significantly with dose, reflecting the dissociation of water molecules by ionizing radiation and the subsequent formation of reactive species (ions and free radicals). These species initiate co-monomer polymerization, leading to the formation of high-molecular-weight polymers within the PVA gel matrix. Greater radiation exposure therefore results in enhanced polymerization, which reduces molecular mobility of water molecules in the vicinity of the polymer and increases the spin-spin relaxation rate (R_2_) [5], as shown in Figure 1a,b. Compared with other polymer gel dosimeters, such as N-vinylpyrrolidone gels [29], the ACAGLPVA formulation exhibits an extended linear dose range (0–30 Gy), though with slightly lower dose sensitivity, as determined from the slope of the linear fit in Figure 1b.

**Figure 3:**
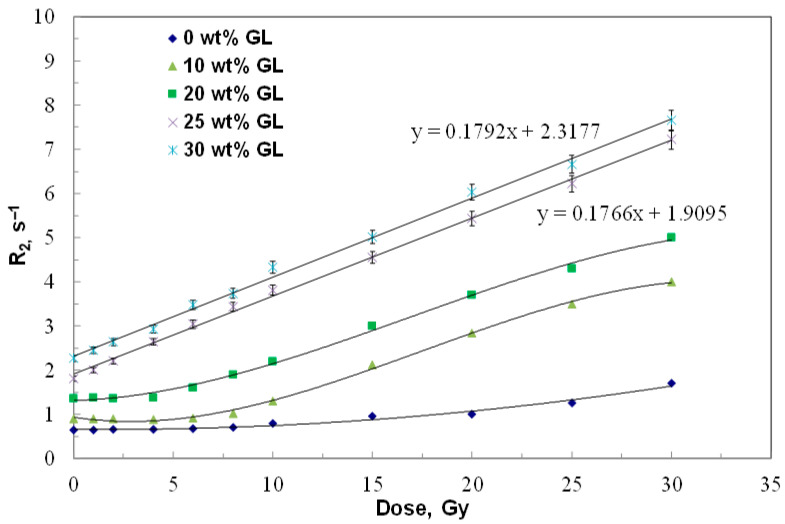
Effect of glucose concentration on dose–response characteristics of the ACAGLPVA gel dosimeter. Transverse relaxation rate (R_2_) versus absorbed dose for formulations containing 0, 10, 20, 25, and 30 wt% glucose (GL). Linear fits are shown only for 25 wt% and 30 wt% glucose formulations, which exhibit complete linear response across the 0–30 Gy range. Error bars represent 2σ of R_2_ values from three independent samples.

Linear fitting was applied to the dose range (0–30 Gy) where the gel exhibits linear dose–response behavior, as this region is most relevant for clinical dosimetry applications in radiotherapy. The non-linear behavior observed at higher doses (30–60 Gy in Figure 1a) reflects monomer depletion and saturation effects, where the available monomers become depleted and the polymerization rate decreases. This phenomenon is common in polymer gel dosimeters at high doses. Similarly, in Figure 3, linear fitting was applied only to gel formulations with optimal glucose concentrations (25 and 30 wt%) that demonstrated complete linear dose–response across the entire 0–30 Gy range. Lower glucose concentrations show non-linear behavior even at low doses, indicating that the sensitizer concentration is insufficient to maintain consistent polymerization kinetics throughout the dose range.

Importantly, unlike conventional gels that require refrigeration to avoid melting and diffusion effects, the ACAGLPVA gel remains stable at ambient temperature, as previously reported [51,53], facilitating both scanning and storage under similar conditions. Furthermore, its dose sensitivity surpasses that of widely used PAGAT dosimeters [54]. Representative photographs of non-irradiated and irradiated gels are shown in Figure 2. As visible in Figure 2, the NMR tubes were not completely filled with polymer gel, leaving an air headspace above the gel surface. The top surface layer of the gel (approximately 1–2 mm) shows minimal color change even after irradiation, in contrast to the bulk gel region, which exhibits dose-dependent color intensity. This phenomenon is due to oxygen inhibition of radiation-induced polymerization at the gel-air interface. Although THPC was employed as an antioxidant to eliminate dissolved oxygen from the bulk gel, oxygen from the air headspace gradually dissolves into the top layer during storage and irradiation. Oxygen acts as a free radical scavenger, terminating the polymerization chain reaction initiated by ionizing radiation. Consequently, the uppermost region remains unpolymerized or shows significantly reduced polymerization. This oxygen-inhibited layer does not affect the dosimetric measurements because the NMR measurements are performed on the bulk gel region well below the surface, and the positioning of samples during irradiation ensures the measurement region is in the oxygen-free zone.

To determine the optimal concentration of glucose (GL) as sensitizer, multiple batches of ACAGLPVA polymer gel dosimeters were prepared with varying GL concentration of 0, 10, 20, 25, and 30 wt%. The gel samples were irradiated with various absorbed doses ranging from 0 to 30 Gy, under the same conditions as outlined in Figure 1. The dose–response characteristics of all polymer gel compositions are illustrated in Figure 3. The results indicate that the dose–response of gel samples with GL concentrations ranging from 0 to 20 wt% did not demonstrate a full linear relationship in the low dose range.

Even though there is an improvement in the linearity of these gel compositions as well as their dose sensitivity with increasing GL concentration from 0 to 20 wt%, the amount of sensitizer is still not enough to speed up the polymerization process in the low dose region. A complete linear dose–response with high dose sensitivity is shown for gel samples that contain a higher amount of GL (i.e., 25 and 30 wt%), implying that the concentration of the sensitizer agent has reached its suitable concentration. The sensitivity of the gel sample containing 25 wt% GL is comparable to that of the 30 wt% sample; hence, 25 wt% has been selected as the optimal GL concentration for this study.

The quantitative dosimetric performance indicators for all glucose concentrations tested are summarized in Table 1. The data clearly demonstrate that glucose concentration significantly affects both the linearity range and dose sensitivity of the ACAGLPVA dosimeter. The optimal formulation containing 25 wt% GL exhibits a dose sensitivity of 0.177 ± 0.005 s^−1^ Gy^−1^ with an extended linear dose range of 0–30 Gy (R^2^ = 0.998). In comparison, the glucose-free formulation (0 wt% GL) shows significantly lower sensitivity (0.070 ± 0.002 s^−1^ Gy^−1^) and a narrow linear range (20–30 Gy), confirming the critical role of glucose in enhancing the dosimetric properties.

Established dosimeters, such as PAGAT, exhibit a linear dose range of 0–20 Gy with a dose sensitivity of 0.09 s^−1^ Gy^−1^ [54]. This indicates that the optimal ACAGLPVA gel containing 25 wt% GL (refer to Table 1) possesses both a higher linear dose range (0–30 Gy) and superior dose sensitivity (0.177 ± 0.005 s^−1^ Gy^−1^) compared to the PAGAT gel dosimeter. Specifically, ACAGLPVA demonstrates a 50% extension in linear dose range and approximately 97% improvement in dose sensitivity relative to PAGAT, representing a significant advancement in polymer gel dosimetry for radiotherapy applications.

**Figure 4:**
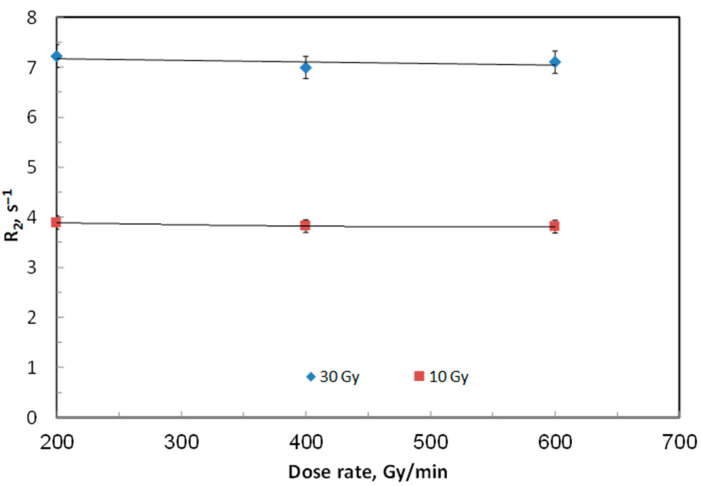
Dose rate independence of ACAGLPVA gel dosimeter. Transverse relaxation rate (R_2_) was measured at doses of 10 Gy (circles) and 30 Gy (squares) as a function of dose rate ranging from 200 to 600 cGy/min. Each data point represents the average of three independent samples, with error bars showing 2σ of R_2_ values.

At a fixed beam energy of 6 MV, the effect of dose-rate on ACAGLPVA gel performance was evaluated within the range of 200–600 cGy/min. Gel samples were irradiated with doses of 10 and 30 Gy under the same conditions used in Figure 1. Each selected dose was administered to a batch of three gel samples, and the average dose response was reported in terms of R_2_ values. As illustrated in Figure 4, no significant variation in relaxation rate was observed across the tested dose rates. This stability suggests that ACAGLPVA gels can be reliably utilized for dose verification and mapping without the need for correction factors related to dose rate. This conclusion aligns with several previous studies indicating that polymer gel dosimeters exhibit independence from dose rate [21].

**Figure 5:**
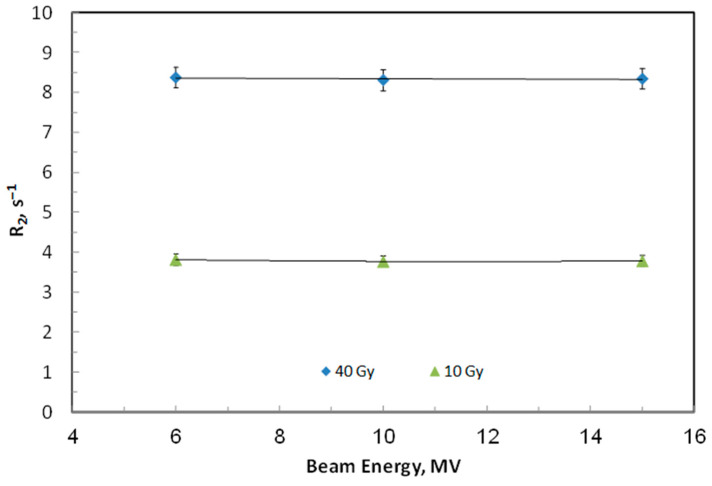
Energy independence of ACAGLPVA gel dosimeter. Transverse relaxation rate (R_2_) was measured at doses of 10 Gy (circles) and 40 Gy (squares) as a function of photon beam energy ranging from 6 to 15 MV. Each data point represents the average of three independent samples, with error bars showing 2σ of R_2_ values.

The effect of radiation beam energy on the dose response of ACAGLPVA was also assessed. Samples were irradiated with doses of 10 and 40 Gy at commonly used radiotherapy energies (6–15 MV) with a fixed dose rate of 600 cGy/min and were characterized using NMR relaxometry one day post-irradiation. As illustrated in Figure 5, the average relaxation rates from triplicate samples showed no significant variation with beam energy. The minor fluctuations in the dose response were within the margin of error, confirming that the performance of the ACAGLPVA dosimeter is stable and independent of photon energy within this range.

**Figure 6:**
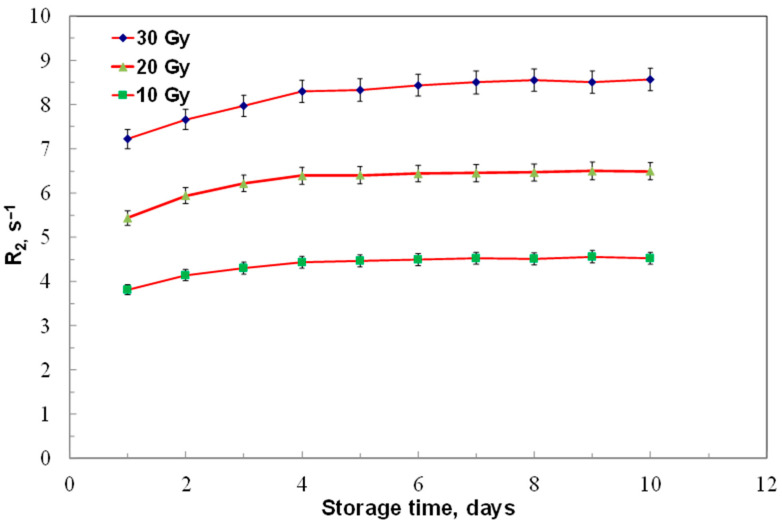
Post-irradiation stability of ACAGLPVA gel dosimeter. Transverse relaxation rate (R_2_) was measured as a function of storage time up to 10 days for samples irradiated to 10, 20, and 30 Gy. A noticeable increase in R_2_ is observed during the first 2–3 days post-irradiation, attributed to continued polymerization, after which the values stabilize. Each data point represents the average of three independent samples, with error bars showing 2σ of R_2_ values.

To assess stability, ACAGLPVA dosimeters irradiated to 10, 20, and 30 Gy were read out over 10 days, with average relaxation rates from three samples shown in Figure 6.

The results demonstrate a noticeable increase in dose response during the first four days (maximum coefficient of variation about 0.07), likely due to ongoing post-irradiation polymerization, in line with earlier reports [52]. After day four, no significant changes were observed up to day ten (maximum coefficient of variation about 0.01), indicating the absence of ongoing polymerization processes caused by long-lived radicals. Quantitatively, for 10 Gy samples, the R_2_ values increased from 3.9 s^−1^ at day 1 to 4.5 s^−1^ at day 4 (15.4% increase), then stabilized at approximately 4.6 s^−1^ from day 4 to day 10 (variation < 5% after day 4). Similar trends were observed for 20 Gy samples and 30 Gy samples. Based on this numerical analysis, we define an optimal reading window of 4–10 days post-irradiation, with maximum stability achieved between days 4–7 where signal drift is less than 5%. For practical applications in radiotherapy dose verification, we recommend conducting readouts at one day post-irradiation, with measurements also being acceptable within a few hours or after four days, as these times correspond to the period of maximum post-irradiation stability.

**Figure 7:**
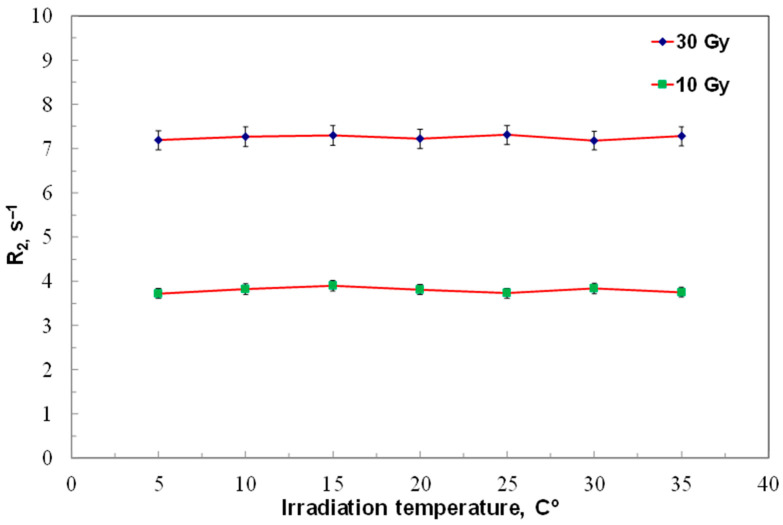
Temperature independence of ACAGLPVA gel dosimeter during irradiation. Transverse relaxation rate (R_2_) was measured at doses of 10 and 30 Gy as a function of irradiation temperature ranging from 5 to 35 °C. Each data point represents the average of three independent samples, with error bars showing 2σ of R_2_ values.

The effect of irradiation temperature on ACAGLPVA dosimeter performance was evaluated at doses of 10 and 30 Gy across 5–35 °C. Three samples were irradiated at each temperature, and average results are shown in Figure 7. The findings demonstrate that the dose response of ACAGLPVA is independent of irradiation temperature within this range, confirming its suitability for dose verification and mapping under varied thermal conditions. This temperature independence results from the dominance of radiation-induced free radical generation over thermal effects, as the activation energy for polymerization in normoxic polymer gels is sufficiently low that temperature variations within 5–35 °C do not significantly affect the dose–response relationship. This behavior contrasts with Fricke-based gel dosimeters, where temperature significantly affects oxidation kinetics [15], and provides a practical advantage for clinical applications by eliminating the need for strict temperature control during gel preparation, irradiation, and storage.

The comprehensive dosimetric performance indicators for the optimal ACAGLPVA formulation (25 wt% GL) are summarized in Table 2. The dosimeter demonstrates excellent performance across all evaluated parameters, including extended linear dose range (0–30 Gy), high dose sensitivity (0.177 ± 0.005 s^−1^ Gy^−1^), dose rate independence (200–600 cGy/min, <2% variation), beam energy independence (6–15 MV, <3% variation), good temporal stability (1–7 days, <5% variation), and temperature independence during irradiation (10–30 °C, <3% variation). These characteristics make ACAGLPVA suitable for clinical radiotherapy dosimetry applications.

It should be noted that the R_2_ measurements in this study were performed using a 0.5 T NMR system. Clinical MRI scanners typically operate at higher field strengths (1.5 T or 3 T). The relationship between R_2_ and magnetic field strength in polymer gel dosimeters is complex and depends on the gel composition and polymer microstructure. Some studies have shown that R_2_ values can increase with higher magnetic field strengths in certain formulations [13], though this behavior is composition-dependent. Since our ACAGLPVA formulation represents a novel composition incorporating glucose as an organic additive, comprehensive characterization at clinically relevant field strengths (1.5 T and 3 T) is necessary to determine the dose sensitivity, linearity range, and stability characteristics under clinical imaging conditions. Future work will focus on this important aspect to facilitate the translation of this dosimeter to clinical practice.

The reproducibility of the ACAGLPVA formulation was assessed through the preparation of four independent batches of the optimal formulation (containing 25 wt% glucose). Each batch was used for different experimental conditions: Batch 1 for glucose concentration optimization (Figure 3), Batch 2 for dose rate dependence (Figure 4), Batch 3 for beam energy dependence (Figure 5), and Batch 4 for irradiation temperature dependence (Figure 7). The excellent reproducibility of the formulation is evidenced by the close agreement of R_2_ values between different batches under similar conditions. For example, comparing the R_2_ values at 20 °C irradiation temperature between Figure 3 (Batch 1) and Figure 7 (Batch 4) demonstrates excellent batch-to-batch consistency, confirming the robustness of our formulation protocol.

## 3. Conclusions

In this study, we successfully developed and characterized a novel ACAGLPVA polymer gel dosimeter incorporating glucose as an organic sensitizer in a PVA-based matrix, representing the first systematic investigation of glucose in PVA-based gel dosimetry. The dosimeter exhibits a linear dose response over the range of 0–30 Gy with excellent linearity (R^2^ = 0.998) and a dose sensitivity of 0.177 ± 0.005 s^−1^ Gy^−1^, which demonstrates superior performance compared to conventional PAGAT dosimeters in terms of both sensitivity and linear dose range. The optimal glucose concentration was determined to be 25 wt%, providing the best combination of sensitivity and linearity. The ACAGLPVA dosimeter demonstrates independence from dose rate (200–600 cGy/min, variation <2%), beam energy (6–15 MV photons, variation <3%), and irradiation temperature (5–35 °C, variation <3%), making it suitable for a wide range of clinical radiotherapy applications. Post-irradiation stability analysis revealed ongoing polymerization during the first four days (coefficient of variation ~0.07), followed by excellent stability from day 4 to day 10 (coefficient of variation ~0.01), defining an optimal reading window of 4–10 days post-irradiation with maximum accuracy achieved at days 4–7.

The ACAGLPVA dosimeter is particularly well-suited for verification of complex dose distributions in advanced radiotherapy techniques such as intensity-modulated radiation therapy (IMRT), volumetric modulated arc therapy (VMAT), and stereotactic radiosurgery, where accurate 3D dose mapping is essential. The room temperature stability of the PVA-based system offers practical advantages over gelatin-based dosimeters that require refrigeration, with the gel setting at room temperature, simplifying clinical handling and eliminating the need for specialized storage conditions.

However, several limitations should be acknowledged. First, the current study was conducted using a 0.5 T NMR system, and further characterization at clinically relevant field strengths (1.5 T or 3 T) is needed to fully establish the dosimeter’s performance in clinical MRI scanners, as R_2_ relaxation rates are field-dependent and may affect dose sensitivity and linearity. Second, the post-irradiation instability during the first four days requires that measurements be delayed until days 4–7 for maximum accuracy, which may not be suitable for applications requiring immediate dose verification, although this is typical of polymer gel dosimetry systems due to ongoing post-irradiation polymerization. Third, while the ACAGLPVA gel composition outperforms other conventional gels within the 0–30 Gy range, the non-linear response above 30 Gy limits the dosimeter’s applicability to high-dose scenarios such as stereotactic body radiotherapy (SBRT) or brachytherapy boost treatments. Additionally, the spatial resolution and 3D dose distribution mapping capabilities have not been quantitatively evaluated in this study, and clinical validation in actual treatment geometries is required before routine clinical implementation.

Future work should focus on: (1) characterizing the ACAGLPVA dosimeter at higher magnetic field strengths (1.5 T and 3 T), (2) optimizing the formulation to improve early post-irradiation stability and potentially extend the linear dose range beyond 30 Gy, (3) quantitatively evaluating spatial resolution and 3D dose mapping accuracy, and (4) validating the dosimeter’s performance in clinical treatment scenarios using advanced 3D imaging modalities such as MRI and optical CT. Despite these limitations, the ACAGLPVA gel dosimeter demonstrates excellent dosimetric properties and represents a significant advancement in polymer gel dosimetry for radiation therapy applications.

## 4. Materials and Methods

### 4.1. Dosimeter Preparation

The acrylic acid–glucose PVA (ACAGLPVA) polymer gel dosimeter was fabricated under standard conditions. All chemicals—ACA, N,N-methylene-bis-acrylamide (BIS), polyvinyl alcohol (PVA), D-(+)-glucose (GL), glutaraldehyde (GTA), and tetrakis(hydroxymethyl)phosphonium chloride (THPC)—were obtained from Sigma-Aldrich, Darmstadt, Germany. An aqueous GL solution was first prepared by dissolving 25 wt% GL powder in 65.7 g of triple-distilled water at room temperature, followed by stirring for 10 min to achieve clarity. The solution was heated to 80 °C, after which 5 wt% PVA was added and stirred for 2 h. The temperature was then reduced to 50 °C to sequentially add 3 wt% BIS (60 min), 0.5 wt% ACA (5 min), 0.5 wt% GTA (3 min), and 0.3 wt% THPC (3 min). THPC serves as an oxygen scavenger, reacting with dissolved oxygen in the gel matrix to prevent oxygen inhibition of radiation-induced polymerization. The use of THPC allows the gel to be prepared under normal atmospheric conditions (ambient air) without requiring an oxygen-free environment, simplifying the preparation protocol while maintaining dosimetric performance. The resulting homogeneous solution was cooled to 35 °C and transferred into airtight 10 mm NMR tubes (Wilmad-LabGlass, Vineland, NJ, USA) to minimize oxygen diffusion from the surrounding air after preparation. The ACAGLPVA samples were stored at room temperature before and after irradiation [51]. The complete composition of the ACAGLPVA gel dosimeter is summarized in Table 3.

Two types of control samples were prepared for this study. First, glucose-free control gels (0 wt% GL) were prepared following the same protocol described above but without glucose addition, with the water content adjusted to 90.7 wt% to maintain the same total mass. These glucose-free controls were used to evaluate the effect of glucose concentration on dose response and sensitivity (Figure 3). Additionally, intermediate glucose concentrations (10, 20, and 30 wt% GL) were prepared to determine the optimal glucose concentration. Second, non-irradiated reference samples were prepared for each gel formulation (including all glucose concentrations tested) and stored under identical conditions (room temperature, dark) as the irradiated samples. These non-irradiated controls were measured using the same NMR protocol to establish baseline R_2_ values (displayed at 0 Gy in Figure 3) and to assess any background polymerization or gel aging effects in the absence of radiation exposure. All control samples were prepared in triplicate and measured at the same time points as the irradiated samples to ensure consistency.

### 4.2. Dosimeter Irradiation

ACAGLPVA gel samples were conditioned at room temperature for five hours prior to irradiation to ensure thermal equilibrium with the irradiation environment. A medical linear accelerator (Varian Medical Systems, Palo Alto, CA, USA) was employed to irradiate the ACAGLPVA samples with doses ranging from 1 to 60 Gy using 6-MV photon beams at a dose rate of 600 cGy/min. The irradiations were conducted in a water phantom (30 × 30 × 30 cm^3^) at a depth of 5 cm, utilizing a 10 × 10 cm^2^ field size and a 100 cm source-to-surface distance. To guarantee the gel samples are irradiated in the beam’s flatness region as specified in the treatment planning system, each ACAGLPVA gel sample was positioned at coordinates 0 × 0 cm^2^.

Some gel samples were exposed to dose rates ranging from 200 to 600 cGy/min to examine the effect of varying dose rates, while keeping the photon beam energy constant at 6 MV. Other samples were irradiated using different beam energies (6–15 MV) at a fixed dose rate of 600 cGy/min to explore how radiation beam energy influences the dose response of the developed gel dosimeter. For accuracy, three samples were irradiated for each dose, and the averaged dose–response values are presented in the figures. Following irradiation, the samples were stored in the dark at room temperature.

### 4.3. Dosimeter Measurement

Both irradiated and non-irradiated ACAGLPVA samples, contained in NMR tubes, were analyzed using a 0.5 T NMR system (Minispec mq20, Bruker, Rheinstetten, Germany). Relaxation rates (R_2_) were measured with a Multi-Spin-Echo Carr–Purcell–Meiboom–Gill (CPMG) sequence, employing 0.5 ms echo spacing, 2000 echoes and 3 s repetition time. The R_2_ values were determined by fitting the multi-echo decay curves to a monoexponential model using the instrument’s built-in software. To minimize the effect of temperature variation during scanning, a thermostatic water bath (Julabo, Seelbach, Germany) maintained the NMR probe temperature at 20 °C ± 0.1 °C. One hour prior to the NMR measurements, the hydrogel samples were placed in a thermostatic circulating water bath to acclimate to the carefully regulated and stable temperature. Each sample was scanned three times, and the median values are reported in the figures.

## Figures and Tables

**Table 1:** Dosimetric Performance Indicators of ACAGLPVA Polymer Gel with Different Glucose Concentrations. The highlighted gel composition is optimal ACAGLPVA gel containing 25 wt% GL.

Gel Composition	Linearity Dose Range (Gy)	Dose Sensitivity (s^−1^ Gy^−1^)	R^2^ Value
ACAGLPVA with 0 wt% GL	20–30	0.070 ± 0.002	0.992
ACAGLPVA with 10 wt% GL	8–30	0.138 ± 0.004	0.995
ACAGLPVA with 20 wt% GL	4–30	0.142 ± 0.005	0.996
**ACAGLPVA with 25 wt% GL**	**0–30**	**0.177 ± 0.005**	**0.998**
ACAGLPVA with 30 wt% GL	0–30	0.179 ± 0.006	0.997

**Table 2:** Summary of Dosimetric Performance Indicators for ACAGLPVA Gel Dosimeter (25 wt% GL).

Performance Indicator	Value/Range	Figure Reference
Dose sensitivity	0.177 ± 0.005 s^−1^ Gy^−1^	Figure 1b and Figure 3
Linear dose range	0–30 Gy (R^2^ = 0.998)	Figure 1a,b
Dose rate independence	200–600 cGy/min	Figure 4
Beam energy independence	6–15 MV photons	Figure 5
Temporal stability	1–7 days post-irradiation	Figure 6
Temperature independence	10–30 °C (irradiation)	Figure 7
Magnetic field strength	0.5 T NMR	Section 4.3
Gel setting	Room temperature, ~10 h	Section 4.1

**Table 3:** Composition of ACAGLPVA Polymer Gel Dosimeter.

Component	Chemical Name/Description	Concentration (wt%)
Water	Triple-distilled water	65.7
GL	D-(+)-Glucose	25.0
PVA	Polyvinyl alcohol	5.0
BIS	N,N′-Methylene-bis-acrylamide	3.0
ACA	Acrylic acid	0.5
GTA	Glutaraldehyde	0.5
THPC	Tetrakis(hydroxymethyl)phosphonium chloride	0.3
Total	-	100.0

## Data Availability

The original contributions presented in this study are included in the article. Further inquiries can be directed to the corresponding author.

## References

[1] Merkis M., Puišo J., Adliene D., Laurikaitiene J. (2021). Development and characterization of silver containing free standing polymer films for dosimetry applications. Polymers.

[2] Sagsoz M.E., Korkut O., Gallo S. (2025). Advancements in tissue-equivalent gel dosimeters. Gels.

[3] Maeyama T., Kato A., Mochizuki A., Sato N., Watanabe Y., Mizukami S. (2019). Dose-rate-independent and diffusion-free nanoclay-based radio-fluorogenic gel dosimeter. Sen. Actuators A Phys..

[4] Zhang W., Wang K., Zeng Y., Hu X., Zhang X., Chang S., Zhang H. (2021). Low-diffusion Fricke gel dosimeters with core-shell structure based on spatial confinement. Materials.

[5] Baldock C., De Deene Y., Doran S., Ibbott G., Jirasek A., Lepage M., McAuley K.B., Oldham M., Schreiner L.J. (2010). Polymer gel dosimetry. Phys. Med. Biol..

[6] Maras P., Kozicki M. (2022). Fast isocenter determination using 3D polymer gel dosimetry with kilovoltage cone-beam CT reading and the PolyGeVero-CT software package for linac quality assurance in radiotherapy. Materials.

[7] Anaraki V., Abtahi S.M.M., Farhood B., Ejtemai-Fard M. (2018). A novel method for increasing the sensitivity of NIPAM polymer gel dosimeter. Radiat. Phys. Chem..

[8] Jaszczak M., Sąsiadek-Andrzejczak E., Kozicki M. (2022). Discolouring 3D gel dosimeter for UV dose distribution measurements. Materials.

[9] Salman M.D., Radzi Y.M., Rahman A.A., Oglat A.A., Dheyab M. (2024). Advancements and applications of dosimetry techniques in modern medical radiation therapy: A comprehensive review. J. Radioanal. Nucl. Chem..

[10] Mattea F., Chacón D., Vedelago J., Valente M., Strumia M.C. (2015). Polymer gel dosimeter based on itaconic acid. Appl. Radiat. Isot..

[11] Thiesen J.H., Hepker J.M., Yu W., Pombier K.D., Kearfott K.J. (2021). Preliminary thermoluminescent dosimeter glow curve analysis with automated glow peak identification for LiF:Mg,Ti. Health Phys..

[12] Maryanski M.J., Schulz R.J., Ibbott G.S., Gatenby J.C., Xie J., Horton D., Gore J.C. (1994). Magnetic resonance imaging of radiation dose distributions using a polymer-gel dosimeter. Phys. Med. Biol..

[13] Maryanski M.J., Gore J.C., Kennan R.P., Schulz R.J. (1993). NMR relaxation enhancement in gels polymerized and cross-linked by ionizing radiation: A new approach to 3D dosimetry by MRI. Magn. Reson. Imaging.

[14] Piotrowski M., Maras P., Kozicki M. (2024). On the use of the Fricke-Pluronic F-127 gel dosimeter for radiation isocenter testing of a medical linear accelerator. Materials.

[15] Gallo S., Lizio D., Monti A.F., Veronese I., Brambilla M.G., Lenardi C., Torresin A., Gambarini G. (2020). Temperature behavior of radiochromic poly(vinyl-alcohol)-glutaraldehyde Fricke gel dosimeters in practice. J. Phys. D Appl. Phys..

[16] Gallo S., Pasquale S., Lenardi C., Veronese I., Gueli A.M. (2021). Effect of ionizing radiation on the colorimetric properties of PVA-GTA xylenol orange Fricke gel dosimeters. Dye. Pigment..

[17] Mizukami S., Watanabe Y., Mizoguchi T., Gomi T., Hara H., Takei H., Fukunishi N., Ishikawa K.L., Fukuda S., Maeyama T. (2021). Whole Three-Dimensional Dosimetry of Carbon Ion Beams with an MRI-Based Nanocomposite Fricke Gel Dosimeter Using Rapid *T*_1_ Mapping Method. Gels.

[18] Ibbott G.S., Maryanski M.J., Eastman P., Holcomb S.D., Zhang Y., Avison R.G., Sanders M., Gore J.C. (1997). Three-dimensional visualization and measurement of conformal dose distributions using magnetic resonance imaging of bang polymer gel dosimeters. Int. J. Radiat. Oncol. Biol. Phys..

[19] De Deene Y., Hanselaer P., De Wagter C., Achten E., De Neve W. (2000). An investigation of the chemical stability of a monomer/polymer gel dosimeter. Phys. Med. Biol..

[20] Zhang P., Jiang L., Chen H., Hu L. (2022). Recent Advances in Hydrogel-Based Sensors Responding to Ionizing Radiation. Gels.

[21] Rabaeh K., Eyadeh M. (2023). Optical properties of polymerization N-(3-methoxypropyl) acrylamide polymer gel dosimeters for radiotherapy. Pigment Resin Technol..

[22] Wong P.S., Garwood D.P., Clarke G.D., McColl R.W., Maryanski M.J., Gore J.C. (1996). Three dimensional measurement of dose distributions produced by a robot-mounted linac using magnetic resonance imaging of bang polymer gel dosimeters. Int. J. Radiat. Oncol. Biol. Phys..

[23] Rashidi A., Abtahi S.M.M., Saeedzadeh E., Akbari M.E. (2020). A new formulation of polymer gel dosimeter with reduced toxicity: Dosimetric characteristics and radiological properties. Zeitschr. Med. Phys..

[24] Roed Y., Kadbi M., Wang J., Pinsky L., Ibbott G.S. (2016). Real-time imaging of 3-dimensional dose distributions with polymer gels using a magnetic resonance–guided linear accelerator. Int. J. Radiat. Oncol. Biol. Phys..

[25] Watanabe Y., Mizukami S., Eguchi K., Maeyama T., Hayashi S.I., Muraishi H., Terazaki T., Gomi T. (2019). Dose distribution verification in high-dose-rate brachytherapy using a highly sensitive normoxic N-vinylpyrrolidone polymer gel dosimeter. Phys. Medica.

[26] Johnston H., Hilts M., Carrick J., Jirasek A. (2012). An x-ray CT polymer gel dosimetry prototype: II. Gel characterization and clinical application. Phys. Med. Biol..

[27] Hilts M., Audet C., Duzenli C., Jirasek A. (2000). Polymer gel dosimetry using x-ray computed tomography: A feasibility study. Phys. Med. Biol..

[28] Raj S., Shankar V., Kumar V., Samuel J. (2015). Reduction in post irradiation CT scan time with green tea extract addition for polymer gel dosimetry. Int. J. Radiat. Oncol. Biol. Phys..

[29] Tachibana H., Watanabe Y., Mizukami S., Maeyama T., Terazaki T., Uehara R., Akimoto T. (2020). End-to-end delivery quality assurance of computed tomography–based high-dose-rate brachytherapy using a gel dosimeter. Brachytherapy.

[30] Oldham M., Siewerdsen J.H., Shetty A., Jaffray D.A. (2001). High resolution gel-dosimetry by optical-CT and MR scanning. Med. Phys..

[31] Lee S., Yi J., Park J., Cho S., Shim J., Chang K., Cao Y., Lee S., Huh H., Kim C. (2010). Development of 3D dosimetry system using polymer gel (TENOMAG) and optical-CT scanner in prostate IMRT. Int. J. Radiat. Oncol. Biol. Phys..

[32] Xu Y., Wuu C., Maryanski M.J. (2004). Performance of a commercial optical CT scanner and polymer gel dosimeters for 3-D dose verification. Med. Phys..

[33] Skyt P.S., Petersen J.B., Yates E.S., Poulsen P.R., Ravkilde T.L., Balling P., Muren L.P. (2013). Dosimetric verification of complex radiotherapy with a 3D optically based dosimetry system: Dose painting and target tracking. Acta Oncol..

[34] Mather M.L., Whittaker A.K., Baldock C. (2002). Ultrasound evaluation of polymer gel dosimeters. Phys. Med. Biol..

[35] Rabaeh K.A., Hammoudeh I.M.E., Moftah B., Oglat A.A., Eyadeh M.M., Aldweri F.M., Abdel-Qader A.J., Devic S. (2022). A normoxic acrylic acid polymer gel for dosimetery in radiation therapy. J. Radioanal. Nucl. Chem..

[36] De Deene Y., Vergote K., Claeys C., De Wagter C. (2006). The fundamental radiation properties of normoxic polymer gel dosimeters: A comparison between a methacrylic acid based gel and acrylamide based gels. Phys. Med. Biol..

[37] Abtahi S.M., Aghamiri S.M.R., Khalafi H. (2014). Optical and MRI investigations of an optimized acrylamide-based polymer gel dosimeter. J. Radioanal. Nucl. Chem..

[38] Abtahi S.M. (2016). Characteristics of a novel polymer gel dosimeter formula for MRI scanning: Dosimetry, toxicity and temporal stability of response. Phys. Medica.

[39] Lepag M., Jayasakera P.M., Bäck S.A., Baldock C. (2001). Dose resolution optimization of polymer gel dosimeters using different monomers. Phys. Med. Biol..

[40] Farhood B., Abtahi S.M.M., Geraily G., Ghorbani M., Mahdavi S.R., Zahmatkesh M.H. (2018). Dosimetric characteristics of PASSAG as a new polymer gel dosimeter with negligible toxicity. Radiat. Phys. Chem..

[41] Rabaeh K.A., Basfar A.A., Almousa A.A., Devic S., Moftah B. (2017). New normoxic N-(hydroxymethyl)acrylamide based polymer gel for 3D dosimetry in radiation therapy. Phys. Medica.

[42] Mattea F., Romero M.R., Vedelago J., Quiroga A., Valente M., Strumia M.C. (2015). Molecular structure effects on the post irradiation diffusion in polymer gel dosimeters. Appl. Radiat. Isot..

[43] Hayashi S.-I., Yoshioka M., Usui S., Haneda K., Kondo T., McAuley K., Tominaga T. (2010). A study on the role of gelatin in methacrylic-acid-based gel dosimeters. Radiat. Phys. Chem..

[44] Abtahi S.M.M., Anaraki V., Farhood B., Mahdavi S.R. (2020). Assessment of photon energy and dose rate dependence of U-NIPAM polymer gel dosimeter. Radiat. Phys. Chem..

[45] Hayashi S.-I., Fujiwara F., Usui S., Tominaga T. (2012). Effect of inorganic salt on the dose sensitivity of polymer gel dosimeter. Radiat. Phys. Chem..

[46] Chacón D., Strumia M., Valente M., Mattea F. (2018). Effect of inorganic salts and matrix crosslinking on the dose response of polymer gel dosimeters based on acrylamide. Radiat. Meas..

[47] Hayashi S.-I., Kawamura H., Usui S., Tominaga T. (2018). Influence of magnesium chloride on the dose–response of polyacrylamide-type gel dosimeters. Radiol. Phys. Technol..

[48] Al-Jarrah A.M., Abdul Rahman A., Shahrim I., Razak N.N., Ababneh B., Tousi E. (2016). Effect of inorganic salts and glucose additives on dose–response, melting point and mass density of genipin gel dosimeters. Phys. Medica.

[49] Rabaeh K.A., Eyadeh M.M., Alrub A.J.A. (2025). Enhancement of the dosimetric properties of N-vinyl caprolactam polymer gel dosimeter for clinical practice. Appl. Radiat. Isot..

[50] Eyadeh M.M., Rabaeh K.A., Issa A.S.B., Diamond K.R. (2023). Evaluation of a novel N-(Hydroxymethyl) acrylamide polymer gel dosimeter formulation with organic glucose additive for radiotherapy. Radiat. Meas..

[51] Rabaeh K.A., Moftah B., Moussa A.A., Bani Issa A.S., Al Kafi M.A. (2024). Optical characterization of a new composition of acrylic acid hydrogel dosimeter for quality assurance in radiotherapy treatment. J. Radioanal. Nucl. Chem..

[52] Awad S.I., Rabaeh K.A., Almousa A.A., Al Kafi A., Masad I.S., Moftah B. (2025). Utilizing acrylic acid polymer hydrogel for 3-D quality assurance in CyberKnife radiotherapy. Radiat. Phys. Chem..

[53] Moftah B., Rabaeh K.A., Moussa A.A., Kafi M.A.A., Bani Issa A.S. (2025). Magnetic properties of polymeric acrylic acid hydrogel dosimeter for radiotherapy applications. Sci. Rep..

[54] Pourfallah T.A., Allahverdi M., Zahmatkesh M.H. (2012). Evaluation of the effects of inhomogeneities on dose profiles using polymer gel dosimeter and Monte Carlo simulation in Gamma Knife. Iran. J. Med. Phys..

